# Validation of Size Estimation of Nanoparticle Tracking Analysis on Polydisperse Macromolecule Assembly

**DOI:** 10.1038/s41598-019-38915-x

**Published:** 2019-02-25

**Authors:** Ahram Kim, Wei Beng Ng, William Bernt, Nam-Joon Cho

**Affiliations:** 1 School of Materials Science and Engineering, Nanyang Technological University, 50 Nanyang Avenue, 639798 Singapore, Singapore; 2 Centre for Biomimetic Sensor Science, Nanyang Technological University, 50 Nanyang Drive, 637553 Singapore, Singapore; 3 Particle Characterization Laboratories, Inc. 845 Olive Ave, Suite A, Novato, CA 94945 USA; 4 School of Chemical and Biomedical Engineering, Nanyang Technological University, 62 Nanyang Drive, 637459 Singapore, Singapore

## Abstract

As the physicochemical properties of drug delivery systems are governed not only by the material properties which they are compose of but by their size that they conform, it is crucial to determine the size and distribution of such systems with nanometer-scale precision. The standard technique used to measure the size distribution of nanometer-sized particles in suspension is dynamic light scattering (DLS). Recently, nanoparticle tracking analysis (NTA) has been introduced to measure the diffusion coefficient of particles in a sample to determine their size distribution in relation to DLS results. Because DLS and NTA use identical physical characteristics to determine particle size but differ in the weighting of the distribution, NTA can be a good verification tool for DLS and vice versa. In this study, we evaluated two NTA data analysis methods based on maximum-likelihood estimation, namely finite track length adjustment (FTLA) and an iterative method, on monodisperse polystyrene beads and polydisperse vesicles by comparing the results with DLS. The NTA results from both methods agreed well with the mean size and relative variance values from DLS for monodisperse polystyrene standards. However, for the lipid vesicles prepared in various polydispersity conditions, the iterative method resulted in a better match with DLS than the FTLA method. Further, it was found that it is better to compare the native number-weighted NTA distribution with DLS, rather than its converted distribution weighted by intensity, as the variance of the converted NTA distribution deviates significantly from the DLS results.

## Introduction

Efforts to develop new drugs are not limited to the physicochemical properties of pharmaceuticals. They also include explorations of effective ways to deliver those drugs without compromising efficacy or safety^1–10^. Despite advances in molecular biology research, many drugs still have serious side effects due to the lack of a specific target and correct control release profile, and these side effects limit our ability to design optimal medications for many diseases, including cancer, neurodegenerative diseases and infectious diseases^11–15^. To address this issue, researchers have developed several new modes of drug delivery system (DDS) that have entered clinical practice, including nanoparticles based on polymers, noble metals and lipid based carriers^3,16–19^. The interactions and stability of such materials are strongly dependent on carrier size, whose characterization is crucial in assessing the quality and determining the efficiency of the DDS^20–22^. In particular, chemical modification of nanoparticles is necessary to make them suitable for physiological conditions, and accurate measurement of their size is necessary for quality control^23–25^. This requirement has become more significant as nanoparticles, and their chemical modifications, have been developed for more specific purposes^1,26,27^. Likewise, lipid vesicles, due to their versatile engineering capabilities, have been combined with various therapeutic agents to achieve desired pharmaceutical properties^28,29^. Due to the inherent self-assembly of lipids, validation of their size and distribution is essential to understand the physical properties that directly correlate with drug efficacy. All of these factors highlight the importance of using accurate and precise measurement techniques to characterize the size distribution of biological and synthetic nanoparticle suspensions.

In the analysis of macromolecular assemblies, various techniques are used to measure the physical properties of samples, including imaging^30,31^, separation of particles^32,33^, scattered light^34,35^ and those measurements are related to the size by conversions relying on various physical principles^36^. Direct imaging techniques, including scanning electron microscopy (SEM), transmission electron microscopy (TEM) and atomic force microscopy (AFM), are some of the most popular methods to obtain the topographical size of particles, as well as their shape and texture^31,36–39^ Imaging has been preferred due to its intuitive high-resolution visualization of particles and the minimal influence of artifacts in size determination^36^. However, imaging methods require laborious sample preparation steps, and the sample must be removed from its native or working environment, often resulting in a deformation to the samples. In addition, throughput is limited and limited sampling may result in biased information^36^.

Another strategy is to separate the particles in the sample, creating a spatial macromolecular redistribution in a medium, in which the degree of separation is determined by the mass or volume of the macromolecules and can be converted into their size^36^. This approach is a feature of various techniques, including size exclusion chromatography (SEC), asymmetrical flow field-flow fractionation (AF4) and analytical ultracentrifugation (AUC), which measure differences in the elution, sedimentation or diffusion of particles^33,40,41^. As the particles in the sample are spatially separated depending on their differences in the course of measurement, these techniques can be combined with other size measurement techniques, such as multi-angle light scattering (MALS), to improve the size resolution or measure additional properties, such as molecular weight^42,43^. As the techniques involve separation of the measured sample, they provide more useful size measurements of polydisperse samples but introduce distortion of the sample condition due to the medium used^36^.

Among non-destructive measurement techniques^44^, dynamic light scattering (DLS) is the most widely used due to its simplicity. Upon laser illumination, the intensity of the light scattered by the particles in suspension changes both temporally and spatially depending on the size and weight of the particles and can be converted into size information^34,36^. Despite being a powerful and accessible tool, DLS has several drawbacks due to the inherent limitation of intensity-biased detection^34,45^. DLS determines a particle’s size from fluctuations of the scattered light resulted from the Brownian motion of the particles. The intensity of the scattered light is proportional to the square of the volume of the particle, which makes DLS very sensitive to the presence of large particles^45,46^. Small amounts of large aggregates or dust particles can disturb the size determination if the main population is significantly smaller in size.

To address this problem, other techniques including AFM, SEM, TEM, AF4 and AUC can be used to verify the size determined by DLS^38,47–49^. However, the definition of size measured by one technique can be different from that of the others, which makes the comparison complicated as it requires careful interpretation of the data obtained. In particular, DLS measures the hydrodynamic diameter from the diffusion coefficient of particles in suspension, and this diffusion coefficient is converted into the diameter of an assumed hypothetical sphere that has the same diffusion coefficient^34^. In contrast, SEM, for instance, can obtain a geometric size of particles given by measuring the width of individual particles from the image^36,50^. Recently, a size characterization tool called nanoparticle tracking analysis (NTA) was introduced to acquire the size of particles by determining their diffusion coefficient, meaning that the definition of the size measured by NTA is identical to that of DLS. NTA can be a good method to verify the results of DLS because they measure the same physical property^51,52^. Whereas DLS reads the intensity change of scattered light to find the diffusion coefficient of particles, NTA calculates the diffusion coefficient based on the movements of individual particles in successive optical video images^51,52^. This difference in the detection principles of DLS and NTA results in a difference in the way that they report size, i.e., the quantities of the particles measured by DLS and NTA are weighted by intensity and number, respectively, which makes NTA an excellent technique for verifying DLS results.

Initial studies on NTA focused on the validation of NTA measurements of mono- and multimodal nanoparticle samples and compared the results with DLS^47,49,52,53^. These studies confirmed that NTA provides comparable results in determining the size of mono- and multimodal nanoparticles compared with DLS. The validation was extended to comparisons with AFM^47^, TEM^38^ and AUC^49^ techniques, where the distribution reported by NTA was a good match for those from the techniques on monomodal samples. When the comparison was extended to polydisperse samples such as proteins and vesicles^52,54–56^, the results showed better size resolution in NTA compared with DLS.

While NTA showed better size resolution than DLS on polydisperse samples, the studies did not observe a narrow distribution from NTA on monodisperse samples. The analysis software for NTA used in the previous studies acquired the size distribution by directly converting the displacement of tracks into the size, which is prone to a stochastic error in determining the size from the particle tracking. Although the studies recognized the uncertainty resulting from the stochastic error^47,49,56^, they used the size distributions from the direct conversion due to the lack of methods to mitigate the stochastic error. To solve this issue, Saveyn *et al*. and Walker suggested size estimation methods based on the maximum likelihood estimation (MLE) principle^57,58^. These methods search a narrower distribution that maximizes the likelihood on the acquired track data either by assuming a certain size distribution with a few adjustable parameters or by taking iterative steps, and can successfully recover a narrower size distribution of monomodal samples from NTA track data. Using this strategy to recover a better size distribution from NTA, monomodal reference samples were measured to compare the results with TEM^30^. Further, Kestens *et al*. compared versions 2.3 and 3.0 of NTA software, which use the direct conversion and the iterative approach, respectively^59^, and found that the iterative approach has a better size resolution on mono- and multi-modal reference samples.

Inspired by previous studies^52,57–59^, this study aimed to evaluate NTA results with respect to the size estimation methods for NTA. First, monodisperse reference standards of known size values were measured using NTA and compared with DLS. The size analysis of acquired particle tracks of NTA was processed by direct conversion of the tracks and with the two MLE-based methods, namely finite track length adjustment (FTLA) and the iterative method. The comparison confirmed that both of the two MLE-based methods can recover a narrow size distribution of the monodisperse reference standards. The estimation methods were also applied to estimate the size distribution of polydisperse vesicle samples prepared in various polydispersity conditions to verify if FTLA and the iterative method are applicable for polydisperse samples of unknown size distribution. While the iterative method achieved results comparable with DLS, the results from FTLA deviated from those of DLS as it assumes an arbitrary size distribution that may not be appropriate for polydisperse samples. In addition, the size distribution of NTA acquired with the estimation methods was converted into an intensity-weighted size distribution from its number-weighted distribution to investigate if the conversion gives a better comparison with the DLS results.

## Results and Discussion

### Size Measurement of Polystyrene Latex Nanoparticle Standards

**Figure 1:**
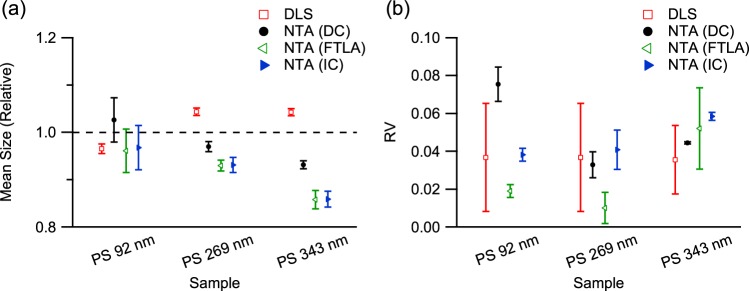
(**a**) Mean and (**b**) relative variance of the size distributions of PS latex nanoparticle samples measured by DLS and NTA. Mean size is displayed as a value relative to its respective nominal sample size. The size information from DLS was acquired using the cumulant method, while the results from NTA were acquired by applying three different methods, i.e., direct conversion of acquired tracks (DC), FTLA and the iterative method (IC).

To validate NTA and its size distribution analysis methods, monodisperse polystyrene (PS) latex standards were measured by DLS and NTA. The mean size and the polydispersity index (PI) of the DLS results were acquired by cumulant analysis of the measured auto-correlation function and are shown in Fig. 1. The mean size obtained by DLS is in good agreement with the nominal values, with 91 ± 1, 278 ± 1 and 352 ± 3 nm acquired for the 92, 269 and 343 nm particle size standards, respectively. The PIs of the samples were 0.037 ± 0.029, 0.016 ± 0.009 and 0.035 ± 0.018, respectively, indicating that the samples were monodisperse.

Size information from NTA, shown in Fig. 1, was extracted from the track data with segment length greater than 5, and processed by the direct conversion method. The mean sizes of the 92, 269 and 343 nm PS standards were 94 ± 4, 261 ± 3 and 320 ± 3 nm, respectively, close to their respective nominal values. The RVs of the 92, 269 and 343 nm PS standards were 0.075 ± 0.009, 0.033 ± 0.007 and 0.044 ± 0.001, confirming that the size distributions were monodisperse.

**Figure 2:**
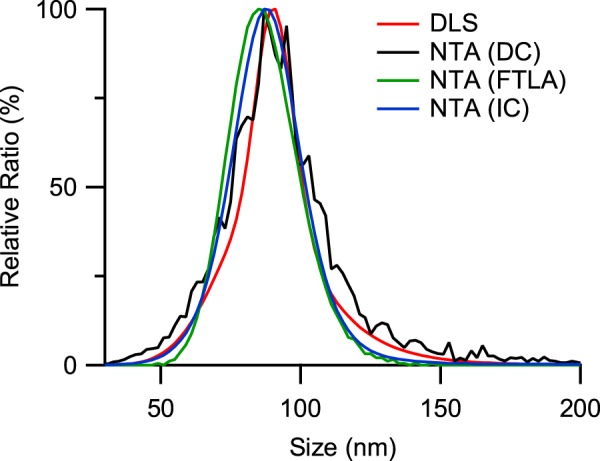
The size distributions of 92-nm PS latex standard measured by DLS and NTA. The size distribution from DLS assumes a log-normal distribution acquired from the cumulant method, while those from NTA are from the three different analysis methods, i.e., direct conversion (DC), FTLA and the iterative method (IC).

To determine if the RV of the NTA results from the direct conversion could be reduced by considering a limited track segment length, the two MLE-based size estimation methods, i.e., FTLA and the iterative method, were applied to the particle tracks acquired from the standard samples. The size distributions of the 92 nm standard sample acquired by direct conversion, FTLA and the iterative method are shown in Fig. 2. The distributions of the other standard samples are presented in the Supporting Information. As shown in Fig. 1, mean sizes of 88 ± 4, 250 ± 3 and 294 ± 7 nm acquired by FTLA and 89 ± 4, 250 ± 4 and 295 ± 6 nm acquired by the iterative method were determined for the 92, 269 and 343 nm standard samples, respectively. RVs of 0.019 ± 0.003, 0.010 ± 0.008 and 0.052 ± 0.022 by FTLA and 0.038 ± 0.003, 0.041 ± 0.010 and 0.058 ± 0.002 by the iterative method were acquired for the 92, 269 and 343 nm standard samples, respectively. The RVs of the 256 and 343 nm standards acquired by the FTLA and iterative methods are not very different from those derived by direct conversion, whereas that of the 92 nm standard is significantly reduced. This suggests that the MLE-based methods are effective for size distribution estimation especially when the particle size is small, which tends to result in a limitation in acquiring tracks of high enough track segment length due to the large diffusion coefficient. For small particle samples, the error in size estimation by direct conversion is enlarged as the error is inversely proportional to the square root of the track segment length, making the observed size distribution broad^57^. For large particle samples, the particles in the sample are tracked for long enough that the error in size estimation by direct conversion is as small as that of the MLE-based methods.

In the comparison, the mean sizes of the standard samples determined by the FTLA and iterative methods match each other well despite their different approaches to finding the maximum likelihood. However, the RVs from FTLA are smaller than the corresponding values from the iterative method. The assumed size distribution of FTLA has the advantage of a smoother size distribution but at the expense of reduced likelihood^60^, resulting in the smaller RVs of these PS standard sample measurements. Given that the samples are monodisperse, assuming a log-normal distribution for the size distribution estimation does not decrease the likelihood compared with the corresponding result from the iterative method (see Supporting Information).

### POPC Vesicles

**Figure 3:**
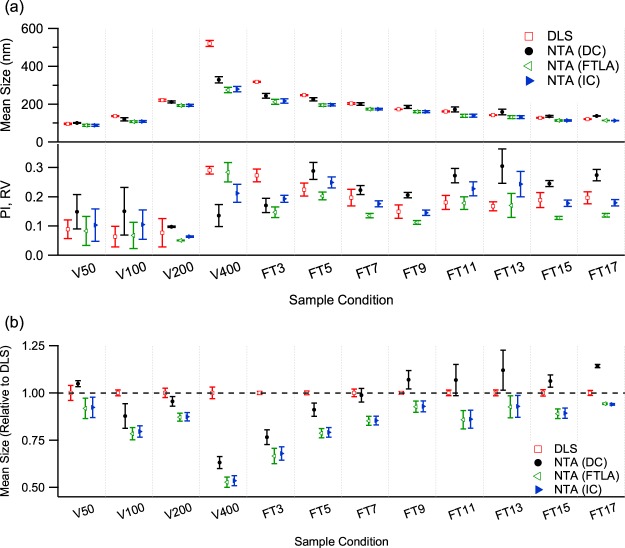
(**a**) Mean and relative variance of the size distributions of the POPC vesicle samples measured by DLS and NTA and (**b**) relative mean size from NTA with respect to their corresponding mean size from DLS. DLS values are derived from the cumulant method, while the NTA results are obtained by applying the three different analysis methods, i.e., direct conversion (DC), FTLA and the iterative method (IC).

POPC vesicles prepared in various polydispersity conditions were measured by DLS. The results are presented in Fig. 3. The mean size of the vesicle samples increased with increasing pore size of the extrusion filter. For vesicle samples extruded through filters with pore sizes of 50 nm (V50), 100 nm (V100), 200 nm (V200) and 400 nm (V400), DLS revealed mean sizes of 91 ± 4, 132 ± 3, 213 ± 4 and 458 ± 13 nm, respectively, and PIs of 0.089 ± 0.032, 0.064 ± 0.035, 0.077 ± 0.048 and 0.291 ± 0.013, respectively. Despite the increase in mean size, the PI does not vary much for pore sizes of 50, 100 and 200 nm, indicating that these samples are relatively monodisperse. However, it jumps to about 0.3 for the 400 nm pore size, showing that the vesicle sample extruded through 400 nm pores is highly polydisperse.

For the vesicles processed by freeze and thaw (FT) treatment before being extruded through a 400-nm pore filter, the mean size shows a gradual decrease with increasing number of cycles. As shown in Fig. 3, for 3, 5, 7, 9, 11, 13, 15 and 17 FT cycles (FT3 to FT17), the mean sizes were 282 ± 4, 223 ± 2, 185 ± 2, 161 ± 1, 148 ± 1, 131 ± 1, 117 ± 2 and 109 ± 1 nm, respectively. The PI results were 0.273 ± 0.021, 0.224 ± 0.022, 0.197 ± 0.029, 0.149 ± 0.023, 0.181 ± 0.024, 0.168 ± 0.015, 0.189 ± 0.025 and 0.197 ± 0.021, respectively, indicating that the polydispersity of the samples reduced upon successive FT cycles but did not decrease enough for the sample to be regarded as monodisperse.

The NTA measurements of the same samples were analyzed by using direct conversion, which determined mean sizes of 100 ± 2, 119 ± 9, 211 ± 6 and 329 ± 17 nm and RVs of 0.149 ± 0.059, 0.151 ± 0.082, 0.097 ± 0.002 and 0.136 ± 0.037 for the vesicle samples extruded through pores of 50, 100, 200 and 400 nm, respectively, and mean sizes of 243 ± 12, 225 ± 9, 201 ± 7, 184 ± 8, 171 ± 13, 158 ± 15, 135 ± 4 and 137 ± 1 nm and RVs of 0.171 ± 0.024, 0.288 ± 0.029, 0.223 ± 0.016, 0.206 ± 0.009, 0.273 ± 0.024, 0.305 ± 0.059, 0.245 ± 0.010 and 0.274 ± 0.019 for the vesicle samples treated by 3, 5, 7, 9, 11, 13, 15 and 17 FT cycles, respectively, before being extruded through a 400 nm filter, as shown in Fig. 3.

For the different extrusion filter pore sizes, the mean sizes measured by NTA were similar to those from DLS, increasing with increasing filter pore size. However, the RVs acquired from NTA indicated that the samples were polydisperse and did not significantly vary with filter pore size, unlike the PI values obtained by DLS. For the various numbers of FT cycles, the trend of mean size measured by NTA also matched that obtained by DLS, decreasing gradually as the number of cycles increased. However, the RVs of the samples from NTA show a gradual increase as the number of FT cycles increases, which is opposite to the trend of the PI measured by DLS, probably because the FT treatment led to homogenization of the vesicle samples^61^. Remarkably, for some of the samples the mean size acquired from NTA was larger than its corresponding mean size from DLS, in contrast to the belief that the mean size of an intensity-weighted distribution is larger than that of its corresponding number-weighted distribution^45,48^. As the polydispersity of the samples is very large, it is more unlikely that a larger mean size will be obtained from NTA.

As direct conversion is not appropriate in determining standard deviation in the measurements of the PS latex standards, the two MLE-based methods were applied for comparison. The results are presented in Fig. 3. Using the FTLA method, NTA obtained mean sizes of 87 ± 5, 106 ± 5, 193 ± 5 and 275 ± 14 nm and RVs of 0.083 ± 0.049, 0.068 ± 0.045, 0.051 ± 0.001 and 0.283 ± 0.033 for the filter pore sizes of 50, 100, 200 and 400 nm, respectively, and mean sizes of 212 ± 13, 195 ± 6, 173 ± 5, 160 ± 5, 138 ± 8, 131 ± 8, 113 ± 3 and 113 ± 1 nm and RVs of 0.147 ± 0.018, 0.203 ± 0.013, 0.135 ± 0.007, 0.112 ± 0.006, 0.178 ± 0.021, 0.170 ± 0.040, 0.128 ± 0.005 and 0.137 ± 0.006 for 3, 5, 7, 9, 11, 13, 15 and 17 FT cycles, respectively. The iterative method determined mean sizes of 88 ± 5, 108 ± 4, 193 ± 5 and 279 ± 14 nm and RVs of 0.103 ± 0.055, 0.105 ± 0.050, 0.064 ± 0.002 and 0.212 ± 0.031 for the filter pore size of 50, 100, 200 and 400 nm, respectively, and mean sizes of 216 ± 11, 196 ± 6, 173 ± 5, 160 ± 5, 138 ± 8, 131 ± 8, 113 ± 4 and 112 ± 1 nm and RVs of 0.193 ± 0.012, 0.249 ± 0.019, 0.176 ± 0.010, 0.145 ± 0.009, 0.228 ± 0.024, 0.243 ± 0.043, 0.178 ± 0.011 and 0.180 ± 0.011 for 3, 5, 7, 9, 11, 13, 15 and 17 FT cycles, respectively. The size distribution profiles of the vesicle samples analyzed by the different methods for NTA measurements are presented in the Supporting Information.

Regardless of the condition of the vesicle samples, the mean sizes of the standard sample measurements estimated by the two MLE-based methods match very well despite the different approaches of the two methods. However, the RVs from the two methods are different, with the RVs from the FTLA method being smaller than those from the iterative method except for the 400-nm filter pore size condition. Although FTLA finds a smaller RV than the iterative method, as noted above, it is at the cost of allowing a smaller likelihood value by assuming a certain shape for the size distribution, i.e., a log-normal distribution, for parameter optimization^60^. In fact, the likelihood value of the size distribution acquired through FTLA is smaller than that from the iterative method except in one case (see Supporting Information). Moreover, some of the likelihood values are even smaller than their corresponding values from the direct conversion, i.e., without any statistical processing. This implies that FTLA is inappropriate for the estimation of size distributions of NTA measurements on polydisperse samples without a proper assumption of the sample size distribution and that the iterative method is preferable to FTLA for such samples^54^.

**Figure 4:**
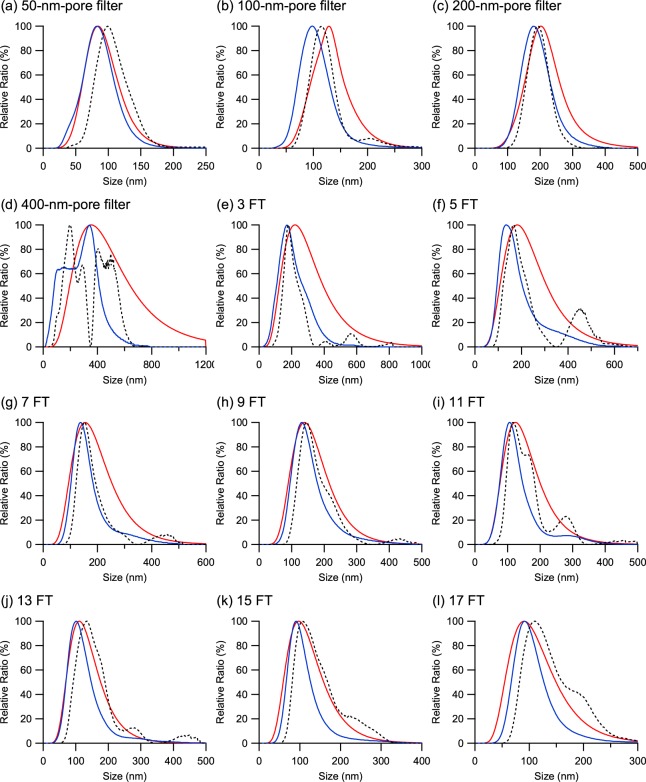
Size distribution of the POPC vesicle samples prepared in various polydispersity conditions measured by DLS and NTA. DLS results (solid red line) are from the cumulant method while NTA results (solid blue line) are acquired by applying the iterative method. For comparison, the size distribution of NTA is reconstructed into an intensity-weighted distribution (dotted black line) by introducing the thin-shell sphere model for the conversion. The vesicle samples were prepared by extrusion through (**a**) 50-nm, (**b**) 100-nm, (**c**) 200-nm and (**d**) 400-nm pore filters or by freeze-thaw treatment of (e-l) 3 to 17 cycles before extrusion through a 400-nm pore filter.

By applying the iterative method as the preferred method for the size distribution estimation of NTA measurements, the size distributions of the vesicle samples acquired by DLS and NTA are compared in Fig. 4. A comparison of the distribution profiles shows that the size distribution of the vesicle samples is monomodal for most of the samples except V400 and the distribution from NTA is shifted toward smaller sizes than its corresponding distribution from DLS. This shift can be attributed to the different physical quantity that the two measurement techniques acquire, i.e., the intensity of scattered light and number of particles for DLS and NTA, respectively, indicating a larger shift for higher polydispersity^45,48^. However, the shift (i.e., the difference in the mean sizes measured by DLS and NTA), is very small compared with the acquired PI and RV of the samples even after applying the iterative method for better size estimation of NTA.

Filipe *et al*. proposed that the small shift can be explained by the lower detection limit of DLS, which detects small particles below 30 nm, which also explains the relatively high PI value measured by DLS^52^. It expects a smaller RV for a size distribution acquired by NTA than the corresponding PI by DLS since NTA would neglect the small size range from the true size distribution and report a narrower distribution. However, the results of FT11 and FT13 show that their respective RV from NTA is larger than their corresponding PI from DLS while the mean size of each sample measured by NTA and DLS is similar, which is contradictory to the expectation.

**Figure 5:**
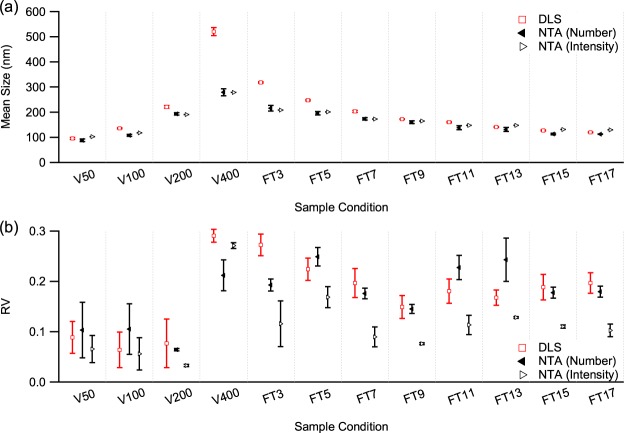
(**a**) Mean size and (**b**) relative variance of the POPC vesicle samples measured by NTA compared with DLS (open red square). NTA results were analyzed by the iterative method (solid black triangle) and reconstructed into an intensity-weighted distribution (open black triangle) using the thin-shell sphere model for the conversion.

Theoretically, the small difference in the mean sizes measured by DLS and NTA can be due to the complex nature of the light scattering intensity in relation to the scattering form factor of the sample^48^. Therefore, the number-weighted size distribution acquired by NTA was reconstructed into an intensity-weighted distribution (as shown in Fig. 4) by assuming an isotropic thin-shell hollow sphere model for the vesicle samples and introducing the RGD approximation to construct the form factor^48,55,62^. The reconstruction revealed mean sizes of 102 ± 1, 118 ± 9, 191 ± 4 and 279 ± 46 nm and RVs of 0.065, 0.056, 0.033 and 0.271 for V50, V100, V200 and V400, respectively, and mean sizes of 209 ± 17, 201 ± 7, 173 ± 4, 165 ± 3, 147 ± 6, 148 ± 5, 131 ± 2 and 130 ± 3 nm and RVs of 0.116 ± 0.045, 0.169 ± 0.021, 0.090 ± 0.020, 0.076 ± 0.002, 0.113 ± 0.019, 0.128 ± 0.002, 0.110 ± 0.003 and 0.102 ± 0.013 for the vesicle samples treated with 3 to 17 FT cycles, respectively. Figure 5 compares the mean sizes and RVs of the original number-weighted and the converted intensity-weighted distributions from NTA with those from DLS. The mean sizes measured by NTA and those acquired from its reconstructed intensity-weighted size distribution are very close to each other. However, the RVs from the reconstructed NTA size distributions are significantly low for some samples, but it is questionable if those samples can be considered monodisperse based only on the reconstructed values. This result indicates that converting the NTA size distribution to an intensity-weighted distribution does not effectively mitigate the difference in the mean sizes observed by DLS and NTA and that it is better to compare the measurements by DLS and NTA when the size distributions are expressed in their original weightings.

## Conclusion

This paper introduces and compares two particle size measurement techniques, DLS and NTA, for the size characterization of polydisperse macromolecular assemblies. While both techniques acquire size information by detecting the diffusion coefficient of the measured particles, they differ in the quantity of the size distribution they report, with NTA reporting number and DLS reporting intensity. Three size distribution estimation methods for NTA were tested to compare their performance using monomodal PS latex standards. The two MLE-based methods, i.e., the FTLA and iterative methods, produced results comparable to each other and in line with those from DLS while the direct conversion resulted in a larger variance, especially for small particles, due to the limitation of obtaining sufficiently long particle tracks. This result indicates that an MLE-based approach should be applied for the accurate measurement of small sized particles with NTA.

The two MLE-based size distribution estimation methods for NTA were further tested on measurements of polydisperse vesicle samples prepared in various polydispersities, which obtained the same mean sizes despite their different strategies to finding the optimal size distribution from the given track data. However, the calculated likelihood of the acquired size distributions obtained by the two methods indicated that FTLA sacrificed likelihood at the expense of a smoother size distribution, further suggesting that the iterative method is preferable for polydisperse samples.

The results for the vesicle samples obtained by NTA using the iterative method were then compared with those from DLS. The mean sizes were comparable except for those samples with a very high polydispersity index, i.e., V400 and FT3. However, the mean sizes of some samples, e.g., FT9 to FT17, measured by NTA were larger than or very close to those from DLS, which seems contradictory to the fact that the mean size from an intensity-weighted size distribution is larger than that from its corresponding number-weighted size distribution. Although the low size detection limit of NTA was pointed out as a source of the contradiction by reducing the variance measured by NTA, it does not fully explain why the mean sizes of V50 were less different between DLS and NTA compared with V100 and V200 despite its higher PI value.

Additionally, the number-weighted size distribution of the vesicle samples from NTA was converted into an intensity-weighted distribution by assuming the thin-shell hollow sphere model with the RGD approximation to verify the influence of the different quantities that DLS and NTA produce. While the mean size given by NTA after conversion was very close to the value before conversion, the RV after the conversion was much reduced compared to that from DLS and NTA. Considering the nature of the vesicle samples, it is questionable whether the small relative variance after reconstruction is reliable. Therefore, the conversion of a number-weighted size distribution of NTA into an intensity-weighted one does not seem to effectively explain the difference in the mean sizes measured by DLS and NTA, and the size information from DLS and NTA is better compared in their original weightings.

## Theory

### Nanoparticle Tracking Analysis

**Figure 6:**
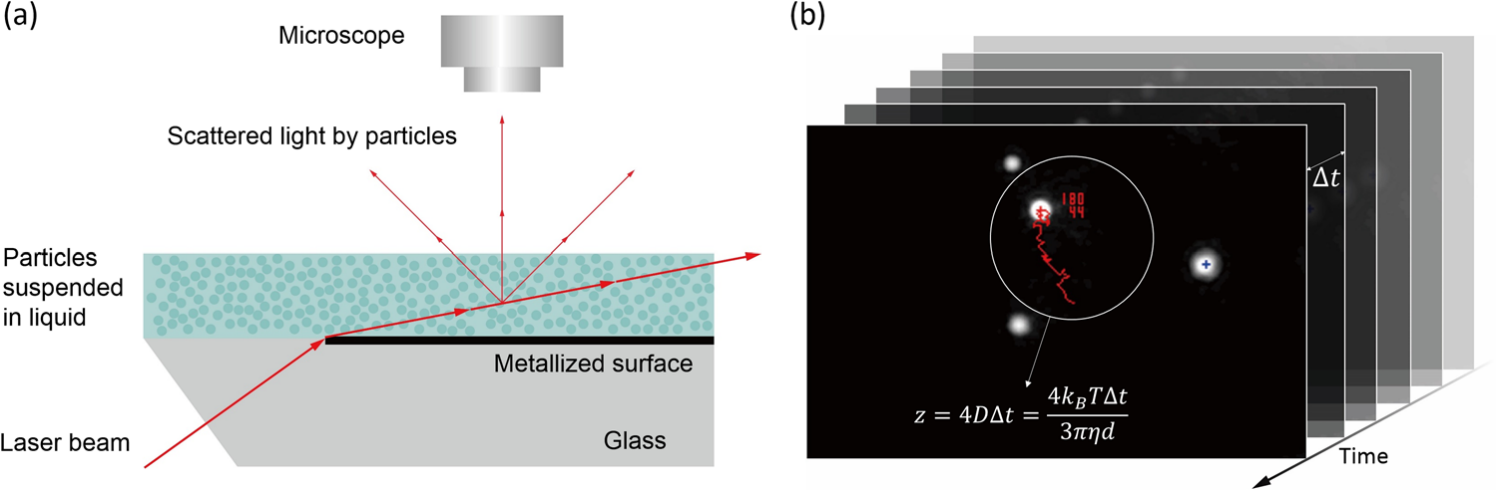
(**a**) Instrumental schematics of NTA detection. The particles suspended in liquid are illuminated by laser beam. Scattered light by the particles is monitored by the microscope, from which the position of the particles is identified by the software. (**b**) The software tracks the trajectory of the particles and constructs particle tracks. The average squared displacement *z* of each track is then converted into the corresponding hydrodynamic diameter *d* according to the Stokes-Einstein equation.

Similar to DLS, NTA extracts the size information of particles in suspension by measuring their diffusion coefficient^51^. As illustrated in Fig. 6, by taking sequential images of illuminated particles in suspension on a periodic time interval, the displacement of a particle can be identified from successive images and constructed into a track. To determine the displacement of particles, NTA compares the two-dimensional location of particles in an image frame with the subsequent frame. In doing so, NTA sets a certain threshold distance, also known as the maximum jump distance, to properly identify if the two particles in the two adjacent frames are the same particle. If any single particle is found in the successive frame within the threshold distance from the location of the particle in the previous image, the two particles are recognized as the same particle and make a track segment. In the same manner, this recognition process is performed for subsequent frames and track segments are combined to construct a particle’s track. The track terminates if there is no particle in the following frame or if there are more than two particles within the threshold distance. Because of the nature of the tracking process, the track segment length, and the number of track segments, are finite and variable.

For a track of a track segment length *n*, the mean squared displacement of the track *z*, expressed as

1 $${z}=\frac{1}{n}\sum _{i=1}^{n}\,{r}_{i}^{2}$$

where *r*_*i*_ is the two-dimensional displacement of *i*th track segment of the track, is translated into the diffusion coefficient assuming a 2D Brownian motion, which is related by

2 $${z}=4{D}{\rm{\Delta }}{t}$$

where *D* is the diffusion coefficient of the tracked particle and Δ*t* is the time interval of the image frames^51,52,57,58^. Then the acquired diffusion coefficient *D* of the track is converted to the hydrodynamic diameter relying on the Stokes-Einstein equation,

3 $$D=\frac{{k}_{B}T}{3\pi \eta d}$$

where *k*_*B*_ is the Boltzmann constant, *T* is the temperature, *η* is the viscosity of the medium and *d* is the hydrodynamic diameter of the tracked particle, and comprises the size distribution of the measured sample.

### Size Distribution Estimation Methods for NTA

#### Uncertainty in the Mean Squared Displacement Measurement in NTA

Measurement of the mean squared displacement *z* in NTA assumes that a particle is tracked for long enough that the measured mean squared displacement is close enough to the ideal mean squared displacement. However, as the track segment length is finite, the acquired mean squared displacement *z* of a track has statistical uncertainty that is inversely proportional to the square root of the track segment length *n*^54,63^. Therefore, small track segment lengths would make the measured size distribution significantly broader than its true size distribution and they should be excluded to have a narrower size distribution.

#### Maximum Likelihood Estimation (MLE) with an Assumed Arbitrary Distribution with Parameters

As particles observed by NTA undergo Brownian motion, the probability distribution of the mean squared displacement *z* of a tracked particle whose size is *d* with a track segment length *n* can be described by a gamma distribution as follows^58^:

4 $$P(z|n,d)=\frac{{(ns)}^{n}}{(n-1)!}{z}^{n-1}\,{e}^{-nsz}$$

where *s* is defined as $1/4D{\rm{\Delta }}t=3\pi \eta d/4{\rm{\Delta }}t{k}_{B}T$. An observed set of tracks Φ of a sample measured by NTA can be related to a particle size distribution *f*(*d*) by the integration

5 $${\rm{\Phi }}={\int }_{0}^{\infty }\,f(x)P(z|n,x)\,dx$$

Although the inversion of the integration would enable recovery of *f*(*d*) by finding *Q*(*d*|*n*, *z*) that satisfies

6 $$f(d)=\sum _{{\rm{\Phi }}}\,{\rm{\Phi }}\cdot Q(d|n,z)$$

the inversion relation cannot be used because *Q*(*d*|*n*, *z*) depends on *f*(*d*)^60^.

**Figure 7:**
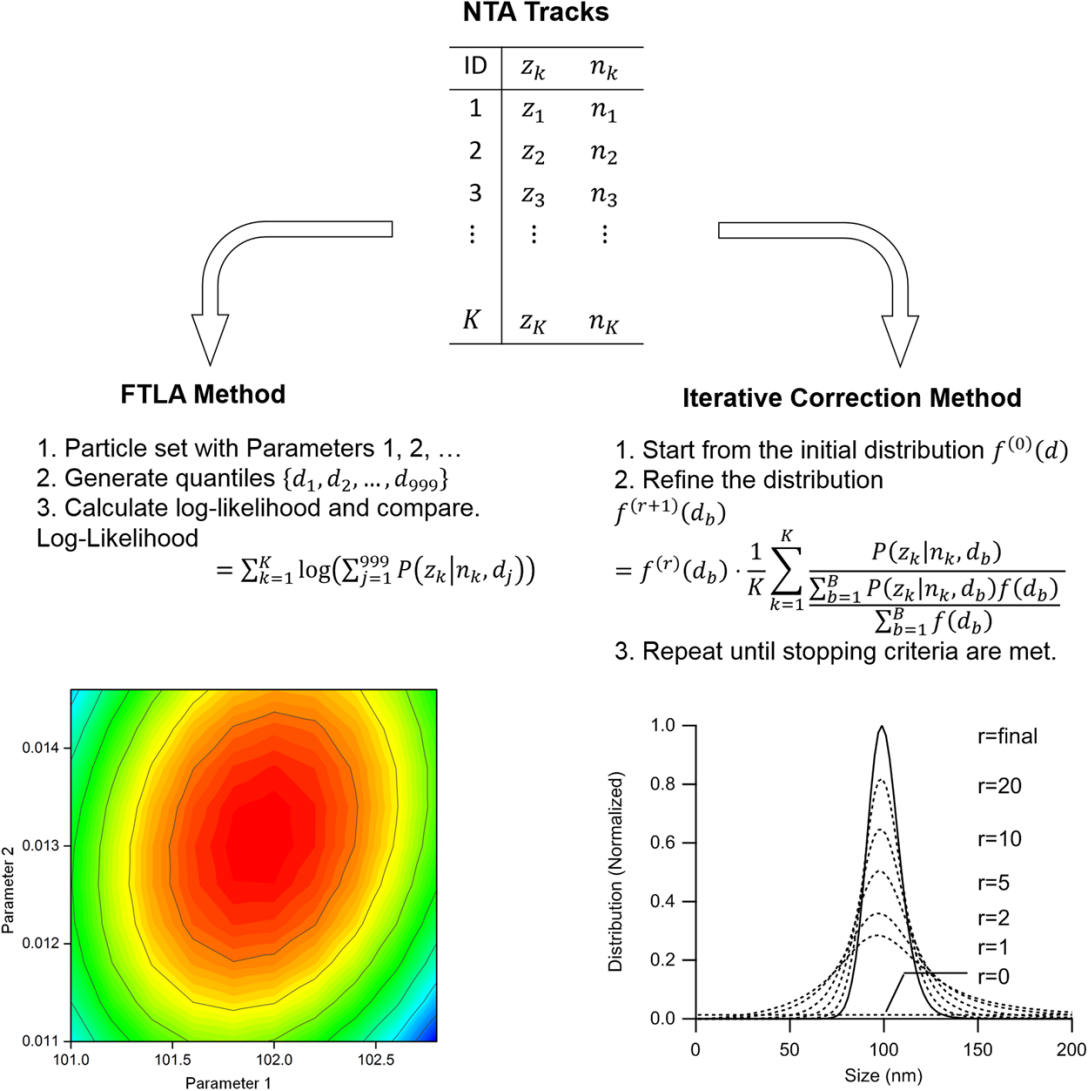
Schematic representation of the size determination methods based on maximum likelihood estimation. By assuming a size distribution described by adjustable parameters, the FTLA method looks for the parameter values that maximize the likelihood of the given NTA tracks. The iterative correction method refines the size distribution that approaches to the maximum likelihood over each iteration.

Due to the difficulty of finding *Q* for the inversion, finite track length adjustment (FTLA) can be used to determine *f*(*d*) by maximizing the likelihood of the observed list of tracks Φ with an assumed size distribution for *f*(*d*) as illustrated in Fig. 7 ^57^. For an assumed size distribution with a few parameters, for example a log-normal distribution with its geometric mean and geometric standard deviation set as parameters, an “ideal” set of particles *d*_*i*_ (with *j* = 1–999) is drawn, which comprises 1000-quantiles of the size distribution, i.e., those of particle size *d*_*i*_ that satisfy *C*(*d*_*j*_) = *j*/1000 (*j* = 1–999) for the cumulative size distribution *C*(*d*) of *f*(*d*). For each particle size *d*_*j*_, the likelihood of producing a track whose mean squared displacement *z* and track segment length *n* is given by

7 $$L(z,n;{d}_{j})=P(z|n,{d}_{j})$$

Hence, the likelihood of producing such a track with a given particle set *d*_*j*_ (*j* = 1–999) is given as

8 $$L(z,n;\{d\})=\sum _{j=1}^{999}P(z|n,{d}_{j})$$

Likewise, the likelihood for all the observed list of tracks Φ becomes

9 $$L({\rm{\Phi }};\{d\})=\prod _{k=1}^{K}(\sum _{j=1}^{999}P({z}_{k}|{n}_{k},{d}_{j}))$$

where *z*_*k*_ and *n*_*k*_ are the mean squared displacement and the track segment length of the *k* th track of Φ, respectively, and *K* is the total number of the observed tracks. For practical reasons, the maximization is performed on the logarithm of the likelihood, as maximizing a positive function also maximizes its logarithm^58^:

10 $$LL({\rm{\Phi }};\{d\})=\,\mathrm{log}(L)=\sum _{k=1}^{K}\,\mathrm{log}(\sum _{j=1}^{999}\,P({z}_{k}|{n}_{k},{d}_{j}))$$

With the log-likelihood, an optimal parameter set for the size distribution is sought for its maximum value, which determines the size distribution for the observed tracks. As this approach assumes an arbitrary size distribution, we can expect a decrease in the likelihood for the benefit of a simpler solution^60^.

#### Maximum Likelihood Estimation by Iterative Correction

For a size distribution *f*(*d*), the log-likelihood of producing the observed list of tracks Φ can be expressed as

11 $$LL({\rm{\Phi }})=\sum _{k=1}^{K}\,\mathrm{log}(\frac{1}{{\sum }_{b=1}^{B}\,f({d}_{b})}\sum _{b=1}^{B}\,P({z}_{k}|{n}_{k},{d}_{b})f({d}_{b}))$$

where *b* is the bin number for the segmented range of particle size *d*^58^.

Then its differentiation with respect to the size distribution is given as

12 $$\frac{\partial LL}{\partial f}=\sum _{k=1}^{K}\frac{P({z}_{k}|{n}_{k},{d}_{b})}{{\sum }_{b=1}^{B}\,P({z}_{k}|{n}_{k},{d}_{b})f({d}_{b})}-\frac{K}{{\sum }_{b=1}^{B}\,f({d}_{b})}$$

At the maximum of the likelihood, the differential becomes zero, leading to

13 $$\sum _{k=1}^{K}\frac{P({z}_{k}|{n}_{k},{d}_{b})}{{\sum }_{b=1}^{B}\,P({z}_{k}|{n}_{k},{d}_{b})f({d}_{b})}=\frac{K}{{\sum }_{b=1}^{B}\,f({d}_{b})}$$

From this relation, it is possible to find the size distribution by an iterative procedure, given as

14 $${f}^{(r+1)}({d}_{b})={f}^{(r)}({d}_{b})\cdot \frac{1}{K}\sum _{k=1}^{K}\frac{P({z}_{k}|{n}_{k},{d}_{b})}{{\sum }_{b=1}^{B}\,P({z}_{k}|{n}_{k},{d}_{b})f({d}_{b})/{\sum }_{b=1}^{B}\,f({d}_{b})}$$

where *f* ^(*r*)^(*d*) is the *r* th estimate of the size distribution from the iterations as illustrated in Fig. 7 ^60^. For the initial size distribution *f* ^(0)^(*d*), a uniform distribution is chosen^58,64,65^.

For the termination criteria of the iteration, the chi-squared statistic of the error between a histogram of the mean squared displacement *H*_*z*_ and that calculated from the *r*th iteration solution *H*^(*r*)^ can be used, where

15 $${\chi }^{2}=\sum _{m=1}^{M}\frac{{({H}_{z}({z}_{m})-{H}^{(r)}({z}_{m}))}^{2}}{{H}^{(r)}({z}_{m})}$$

16 $${H}^{(r)}({z}_{m})=\sum _{n={N}_{{\min }}}^{{N}_{\max }}{N}_{n}\sum _{b=1}^{B}\frac{P({z}_{m}|n,{d}_{b}){f}^{(r)}({d}_{b}){\rm{\Delta }}{z}_{m}}{{\sum }_{b=1}^{B}\,{f}^{(r)}({d}_{b})}$$

where *m* is the bin number for the mean squared displacement and *N*_*n*_ is the number of tracks with track segment length *n*, and in this study the iteration terminates when the change in *χ*^2^ becomes smaller than 1% of the previous value^58^.

### Conversion of Number-Weighted Distribution of NTA

The size distribution measured by DLS is weighted by the intensity of the scattered light, which is dependent on the particle size, whereas that from NTA is weighted by the number. In comparing the two quantities, one must be converted to match the weighting of the other.

The number-weighted size distribution *f*(*d*) from NTA can be converted into an intensity-weighted distribution *f*_*I*_(*d*) given by

17 $${f}_{I}(d)=\frac{f(d)I(q,d)}{{\int }_{0}^{\infty }f(d)I(q,d)dd}$$

where *q* is the scattering vector and *I*(*q*,*d*) is the form factor of the measured particles^66,67^. For vesicles, we assume a thin-shell hollow sphere model^48,68,69^, so that

18 $$I(q,d)={(\pi {d}^{2}t)}^{2}P(q,d)$$

where *P*(*q*,*d*) is the structural factor for the vesicle approximated by the Rayleigh-Gans-Debye (RGD) approximation given by

19 $$P(q,d)={(\frac{\sin (q\cdot d/2)}{q\cdot d/2})}^{2}$$

## Materials and Methods

### Reagents

Polystyrene latex standards (92 ± 3, 269 ± 7 and 343 ± 9 nm) were purchased from Thermo Scientific (Rockford, IL., USA). 1-palmitoyl-2-oleoyl-sn-glycero-3-phosphocholine (POPC) was purchased from Avanti Polar Lipids Inc., (Alabaster, AL, USA).

### Vesicle preparation

Vesicles composed of egg phosphatidylcholine (PC) (Avanti Polar Lipids Inc., Alabaster, AL) were prepared at a lipid concentration of ∼5 mg/mL and then diluted before the experiment. Briefly, dried lipid films were hydrated with 10 mM Tris (pH 7.5) buffer solution with 150 mM NaCl, and the sample was then vortexed periodically for 5 min. Vesicle samples were extruded through polycarbonate membranes with either 50, 100, 200 or 400 nm pores sized by a miniextruder (Avanti Polar Lipids). For those samples pretreated with a range of freeze-thaw cycles, freeze-thaw treatment was performed before extrusion on newly hydrated lipid films by using a previously described methodology based on freeze-thaw pretreatment and then extrusion^61^. Specifically, in each treatment cycle, the vesicle suspension was frozen in liquid nitrogen for 30 s, before thawing in an 80 °C water bath for 90 s, and then finally being vortexed to complete each cycle. After the freeze-thaw cycles of 3, 5, 7, 9, 11, 13, 15 or 17 repetitions, vesicles were sized by an extruder (Avanti Polar Lipids) through 400 nm polycarbonate membrane pores. All aqueous solutions and buffers were prepared in Milli-Q water with a minimum resistivity of 18.2 MΩ·cm (Millipore, Billerica, MA).

### Experimental setup

For the DLS measurements, a ZetaPals particle size analyzer (Brookhaven Instruments, Holtsville, NY) with a 658.0 nm monochromatic laser was used. For each sample, three independent runs of 1 min were performed. To avoid unnecessary reflection, all measurements were taken at a scattering angle of 90°, and the measured intensity autocorrelation function was fitted to yield the intensity-weighted size distribution of particles in solution. The deconvolution of the autocorrelation function was done using the cumulants method, which was applied to calculate the intensity-weighted log-normal profile of the size distribution expressed by the average effective diameter and its polydispersity.

NTA measurements were made with an LM10HS (Nanosight Limited, Amesbury, UK) equipped with a scientific CMOS camera, a 20x objective lens, a blue laser module (405 nm, LM12 version C) and NTA software version 3.1. A 1-ml disposable syringe was used to inject the samples into the instrument chamber. The video data for NTA measurements were collected for 30 seconds, repeated three times for each sample. The detection threshold of the NTA software was set to 5 and the maximum jump distance and the minimum track segment length were both set to auto.

Detected tracks were then translated into a size distribution using three different methods, i.e., direct conversion of the detected particle tracks, maximum likelihood estimation with an assumed distribution (the FTLA method) and maximum likelihood estimation by iterative correction. For the conversion, valid tracks were acquired from the detected tracks by the software, with tracks of a small track segment length excluded from the selection.

FTLA assumes a certain shape for the size distribution to maximize likelihood, and we assumed a log-normal distribution in this study so that the mean and standard deviation parameters could be optimized to produce maximum likelihood^57^. For the calculation, 1000-quantiles are generated from a log-normal distribution while varying its mean size and standard deviation, which represent an “ideal” set of particles for the assumed size distribution^57^.

In the iterative method, the size distribution *f*(*d*) is refined by the iteration

20 $$\begin{array}{rcl}{f}^{(j+1)}({d}_{i}) & = & {f}^{(j)}({d}_{i})\cdot \frac{1}{N}\sum _{k}\,\frac{P({z}_{k};{n}_{k},{d}_{i})}{{Q}^{(j)}}\\ {Q}^{(j)} & = & \sum _{i}\,P({z}_{k};{n}_{k},{d}_{i}){f}^{(j)}({d}_{i})\end{array}$$

where *f* ^(*j*)^(*d*_*i*_) is a normalized fraction of those particles in the size distribution of the *j*th iteration whose size is between *d*_*i*_ and *d*_*i*+1_, and *N* is the number of tracks in the given set of tracks. The initial size distribution *f* ^(0)^(*d*) is set to a uniform distribution so that *f* ^(0)^(*d*) = 1/*M*, where *M* is the number of bins for the size distribution. The iterations are performed until the change in the chi-squared value between the histogram of the displacement of the observed tracks and that of the estimated size distribution from the iteration is less than 1% of the previous value^58^.

In comparing the results from DLS and NTA, the mean size of NTA from the acquired size distribution is compared with the mean hydrodynamic size *R*_*z*_ of DLS while the relative variance (RV, the variance divided by the square of the mean size) of NTA is compared with the PI of DLS.

## Supplementary information

Validation of Size Estimation of Nanoparticle Tracking Analysis on Polydisperse Macromolecule Assembly

## Data Availability

The data that support the findings of this study are present in the paper and the Supplementary Information. Additional information is available from the authors upon reasonable request.

## References

[1] Shi J, Votruba AR, Farokhzad OC, Langer R (2010). Nanotechnology in drug delivery and tissue engineering: from discovery to applications. Nano Lett.

[2] Parveen S, Misra R, Sahoo SK (2012). Nanoparticles: a boon to drug delivery, therapeutics, diagnostics and imaging. Nanomedicine.

[3] Blanco E, Shen H, Ferrari M (2015). Principles of nanoparticle design for overcoming biological barriers to drug delivery. Nat Biotechnol.

[4] Chang HI, Yeh MK (2012). Clinical development of liposome-based drugs: formulation, characterization, and therapeutic efficacy. Int J Nanomedicine.

[5] Tan ML, Choong PF, Dass CR (2010). Recent developments in liposomes, microparticles and nanoparticles for protein and peptide drug delivery. Peptides.

[6] Bamrungsap S (2012). Nanotechnology in therapeutics: a focus on nanoparticles as a drug delivery system. Nanomedicine (Lond).

[7] Warne NW (2011). Development of high concentration protein biopharmaceuticals: the use of platform approaches in formulation development. Eur J Pharm Biopharm.

[8] Tsuchikama K, An Z (2018). Antibody-drug conjugates: recent advances in conjugation and linker chemistries. Protein Cell.

[9] Li BL (2017). Directing Assembly and Disassembly of 2D MoS2 Nanosheets with DNA for Drug Delivery. ACS Appl Mater Interfaces.

[10] Komiyama M, Yoshimoto K, Sisido M, Ariga K (2017). Chemistry Can Make Strict and Fuzzy Controls for Bio-Systems: DNA Nanoarchitectonics and Cell-Macromolecular Nanoarchitectonics. Bull Chem Soc Jpn.

[11] Muller RH, Keck CM (2004). Challenges and solutions for the delivery of biotech drugs–a review of drug nanocrystal technology and lipid nanoparticles. J Biotechnol.

[12] Brigger I, Dubernet C, Couvreur P (2012). Nanoparticles in cancer therapy and diagnosis. Adv Drug Deliv Rev.

[13] Barenholz Y (2012). Doxil^®^, the first FDA-approved nano-drug: Lessons learned. J Control Release.

[14] Cavalli A (2008). Multi-target-directed ligands to combat neurodegenerative diseases. J Med Chem.

[15] Sams-Dodd F (2005). Target-based drug discovery: is something wrong?. Drug Discov Today.

[16] Gregoriadis G (1995). Engineering liposomes for drug delivery: Progress and problems. Trends Biotechnol.

[17] Rawat M, Singh D, Saraf S, Saraf S (2006). Nanocarriers: promising vehicle for bioactive drugs. Biol Pharm Bull.

[18] Al-Jamal WT, Kostarelos K (2011). Liposomes: from a clinically established drug delivery system to a nanoparticle platform for theranostic nanomedicine. Acc Chem Res.

[19] Metselaar JM, Storm G (2005). Liposomes in the treatment of inflammatory disorders. Expert Opin Drug Deliv.

[20] He CB, Yin LC, Tang C, Yin CH (2012). Size-dependent absorption mechanism of polymeric nanoparticles for oral delivery of protein drugs. Biomaterials.

[21] Banerjee A, Qi JP, Gogoi R, Wong J, Mitragotri S (2016). Role of nanoparticle size, shape and surface chemistry in oral drug delivery. J Control Release.

[22] Coradeghini R (2013). Size-dependent toxicity and cell interaction mechanisms of gold nanoparticles on mouse fibroblasts. Toxicol Lett.

[23] Braun NJ, DeBrosse MC, Hussain SM, Comfort KK (2016). Modification of the protein corona-nanoparticle complex by physiological factors. Mater Sci Eng C Mater Biol Appl.

[24] Hama S (2015). Overcoming the polyethylene glycol dilemma via pathological environment-sensitive change of the surface property of nanoparticles for cellular entry. J Control Release.

[25] Natarajan SK, Selvaraj S (2014). Mesoporous silica nanoparticles: importance of surface modifications and its role in drug delivery. RSC Adv..

[26] Soliman GM (2010). Tailoring the efficacy of nimodipine drug delivery using nanocarriers based on A2B miktoarm star polymers. Biomaterials.

[27] Coelho JF (2010). Drug delivery systems: Advanced technologies potentially applicable in personalized treatments. EPMA J.

[28] Love WG, Amos N, Kellaway IW, Williams BD (1990). Specific accumulation of cholesterol-rich liposomes in the inflammatory tissue of rats with adjuvant arthritis. Ann Rheum Dis.

[29] Hua S, Wu SY (2013). The use of lipid-based nanocarriers for targeted pain therapies. Front Pharmacol.

[30] de Temmerman PJ, Verleysen E, Lammertyn J, Mast J (2014). Size measurement uncertainties of near-monodisperse, near-spherical nanoparticles using transmission electron microscopy and particle-tracking analysis. J Nanopart Res.

[31] Troiber C (2013). Comparison of four different particle sizing methods for siRNA polyplex characterization. Eur J Pharm Biopharm.

[32] Fraunhofer W, Winter G, Coester C (2004). Asymmetrical flow field-flow fractionation and multiangle light scattering for analysis of gelatin nanoparticle drug carrier systems. Anal Chem.

[33] Liu J, Andya JD, Shire SJ (2006). A critical review of analytical ultracentrifugation and field flow fractionation methods for measuring protein aggregation. AAPS J.

[34] Berne, B. J. & Pecora, R. *Dynamic light scattering: with applications to chemistry*, *biology*, *and physics*. Dover edn, (Dover Publications, 2000).

[35] Maulucci G (2005). Particle size distribution in DMPC vesicles solutions undergoing different sonication times. Biophys J.

[36] Merkus, H. G. *Particle size measurements: fundamentals*, *practice*, *quality*. (Springer, 2009).

[37] Sokolova V (2011). Characterisation of exosomes derived from human cells by nanoparticle tracking analysis and scanning electron microscopy. Colloids Surf B Biointerfaces.

[38] Anderson W, Kozak D, Coleman VA, Jamting AK, Trau M (2013). A comparative study of submicron particle sizing platforms: accuracy, precision and resolution analysis of polydisperse particle size distributions. J Colloid Interface Sci.

[39] Bootz A, Vogel V, Schubert D, Kreuter J (2004). Comparison of scanning electron microscopy, dynamic light scattering and analytical ultracentrifugation for the sizing of poly(butyl cyanoacrylate) nanoparticles. Eur J Pharm Biopharm.

[40] Striegel, A. M. *Modern size-exclusion liquid chromatography: practice of gel permeation and gel filtration chromatography*. 2nd edn, (Hoboken, N. J.: Wiley, 2009).

[41] Yohannes G, Jussila M, Hartonen K, Riekkola ML (2011). Asymmetrical flow field-flow fractionation technique for separation and characterization of biopolymers and bioparticles. J Chromatogr A.

[42] Wyatt PJ (1993). Light scattering and the absolute characterization of macromolecules. Anal Chim Acta.

[43] Podzimek, S. *Light scattering*, *size exclusion chromatography*, *and asymmetric flow field flow fractionation: powerful tools for the characterization of polymers*, *proteins*, *and nanoparticles*. (Hoboken, N. J.: Wiley, 2011).

[44] van der Pol E, Coumans F, Varga Z, Krumrey M, Nieuwland R (2013). Innovation in detection of microparticles and exosomes. J Thromb Haemost.

[45] Hassan PA, Rana S, Verma G (2015). Making sense of Brownian motion: colloid characterization by dynamic light scattering. Langmuir.

[46] Stetefeld J, McKenna SA, Patel TR (2016). Dynamic light scattering: a practical guide and applications in biomedical sciences. Biophys Rev.

[47] Boyd RD, Pichaimuthu SK, Cuenat A (2011). New approach to inter-technique comparisons for nanoparticle size measurements: using atomic force microscopy, nanoparticle tracking analysis and dynamic light scattering. Colloids Surf A Physicochem Eng Asp.

[48] Hallett FR, Watton J, Krygsman P (1991). Vesicle sizing: Number distributions by dynamic light scattering. Biophys J.

[49] Bell NC, Minelli C, Tompkins J, Stevens MM, Shard AG (2012). Emerging techniques for submicrometer particle sizing applied to Stober silica. Langmuir.

[50] Amini R, Brar SK, Cledon M, Surampalli RY (2016). Intertechnique comparisons for nanoparticle size measurements and shape distribution. J Hazard Toxic Radioact Waste.

[51] Malloy A, Carr B (2006). NanoParticle tracking analysis - The Halo™ system. Part Part Syst Charact.

[52] Filipe V, Hawe A, Jiskoot W (2010). Critical evaluation of nanoparticle tracking analysis (NTA) by NanoSight for the measurement of nanoparticles and protein aggregates. Pharm Res.

[53] James AE, Driskell JD (2013). Monitoring gold nanoparticle conjugation and analysis of biomolecular binding with nanoparticle tracking analysis (NTA) and dynamic light scattering (DLS). Analyst.

[54] Dragovic RA (2011). Sizing and phenotyping of cellular vesicles using nanoparticle tracking analysis. Nanomedicine.

[55] Yang DT, Lu X, Fan Y, Murphy RM (2014). Evaluation of Nanoparticle Tracking for Characterization of Fibrillar Protein Aggregates. AIChE J.

[56] Gross J, Sayle S, Karow AR, Bakowsky U, Garidel P (2016). Nanoparticle tracking analysis of particle size and concentration detection in suspensions of polymer and protein samples: Influence of experimental and data evaluation parameters. Eur J Pharm Biopharm.

[57] Saveyn H (2010). Accurate particle size distribution determination by nanoparticle tracking analysis based on 2-D Brownian dynamics simulation. J Colloid Interface Sci.

[58] Walker JG (2012). Improved nano-particle tracking analysis. Meas Sci Technol.

[59] Kestens V, Bozatzidis V, De Temmerman PJ, Ramaye Y, Roebben G (2017). Validation of a particle tracking analysis method for the size determination of nano- and microparticles. J Nanopart Res.

[60] Lucy LB (1974). Iterative technique for rectification of observed distributions. Astronom J.

[61] Mayer LD, Hope MJ, Cullis PR, Janoff AS (1985). Solute distributions and trapping efficiencies observed in freeze-thawed multilamellar vesicles. Biochim Biophys Acta.

[62] Matsuzaki K (2000). Optical characterization of liposomes by right angle light scattering and turbidity measurement. Biochim Biophys Acta.

[63] Gardiner, C., Ferreira, Y. J., Dragovic, R. A., Redman, C. W. & Sargent, I. L. Extracellular vesicle sizing and enumeration by nanoparticle tracking analysis. *J Extracell Vesicles* **2**, 10.3402/jev.v2i0.19671 (2013). 10.3402/jev.v2i0.19671 PMC3760643 24009893

[64] Veklerov E, Llacer J (1987). Stopping rule for the MLE algorithm based on statistical hypothesis testing. IEEE Trans Med Imag.

[65] Hebert TJ (1990). Statistical stopping criteria for iterative maximum-likelihood reconstruction of emission images. Phys Med Biol.

[66] Hanus LH, Ploehn HJ (1999). Conversion of intensity-averaged photon correlation spectroscopy measurements to number-averaged particle size distributions. 1. Theoretical development. Langmuir.

[67] Patty PJ, Frisken BJ (2006). Direct determination of the number-weighted mean radius and polydispersity from dynamic light-scattering data. Appl Opt.

[68] Pecora R, Aragon SR (1974). Theory of light scattering from hollow spheres. Chem Phys Lipids.

[69] Pencer J, Hallett FR (2003). Effects of vesicle size and shape on static and dynamic light scattering measurements. Langmuir.

