# Supplementary material for: Validation of Size Estimation of Nanoparticle Tracking Analysis on Polydisperse Macromolecule Assembly
Source: Sci Rep. 2019 Feb 25;9:2639. doi: 10.1038/s41598-019-38915-x (PMC6389903; doi:10.1038/s41598-019-38915-x)
Supplement: Supplementary file 1 — Validation of Size Estimation of Nanoparticle Tracking Analysis on Polydisperse Macromolecule Assembly [file 41598_2019_38915_MOESM1_ESM.pdf]

Validation of Size Estimation of Nanoparticle Tracking Analysis on Polydisperse Macromolecule Assembly

Ahram Kim[1], Wei Beng Ng[1,2], William Bernt[3] and Nam-Joon Cho*[1,2,4]

1 School of Materials Science and Engineering, Nanyang Technological University, 50 Nanyang Avenue 639798, Singapore

2 Centre for Biomimetic Sensor Science, Nanyang Technological University, 50 Nanyang Drive 637553, Singapore

3 Particle Characterization Laboratories, Inc. 845 Olive Ave, Suite A, Novato, CA 94945

4 School of Chemical and Biomedical Engineering, Nanyang Technological University, 62 Nanyang Drive 637459, Singapore

Correspondence and requests for materials should be addressed to NJC. (email: njcho@ntu.edu.sg)

Dynamic Light Scattering

DLS extracts the size distribution of particles in suspension from their Brownian motion, whose displacement is related to their diffusion coefficient.[1-3] The intensity of the light scattered by particles in suspension upon laser illumination changes due to the particles' Brownian motion, and the intensity fluctuation is processed to acquire its auto-correlation function whereby the decay time is related to the particles' diffusion coefficient as follows

$$g^{(1)}(\tau) = A \cdot e^{-Dq^2\tau} + B \quad (S1)$$

where $g^1(\tau)$ is the first-order correlation function of the electric field, τ is a delay time, D is the diffusion coefficient, q is the scattering vector and A and B are the amplitude and the baseline of the correlation function, respectively. For monodisperse spherical particles, the hydrodynamic radius R_h can be related to the diffusion coefficient using the Stokes-Einstein equation

$$D = \frac{k_B T}{6\pi\eta R_h} \quad (S2)$$

where k_B is the Boltzmann constant, T is the absolute temperature and η is the viscosity of the medium.

For polydisperse samples, the auto-correlation function can be expressed as the integral form

$$g^{(1)}(\tau) = \int G(\Gamma)\exp(-\Gamma\tau)d\Gamma \quad (S3)$$

where $\Gamma = Dq^2$ is the decay rate for a given size with diffusion coefficient D and $G(\Gamma)$ is the intensity-weighted size distribution. Although a Laplace inversion of the integration produces the size distribution $G(\Gamma)$, it is well known that the inversion is not a well-conditioned problem.[1,3,4] Many strategies have been suggested to recover the size distribution from the integration, among which the cumulant analysis method is only method recommended by the International Standards Organization.[5]

The cumulant analysis method is applied for relatively narrow polydisperse samples[6]. The mean $\bar{\Gamma}$ and the variance μ_2 of the distribution $G(\Gamma)$ are acquired from the expansion of the logarithm of the auto-correlation function as

$$\ln\left(g^1(\tau)\right) = \ln B - \bar{\Gamma}\tau + \frac{\mu_2}{2!}\tau^2 - \frac{\mu_3}{3!}\tau^3 + \cdots \quad \text{(S4)}$$

where

$$\bar{\Gamma} = \int G(\Gamma) d\Gamma$$

$$\mu_2 = \int G(\Gamma)(\Gamma - \bar{\Gamma})^2 d\Gamma$$

$$\mu_3 = \int G(\Gamma)(\Gamma - \bar{\Gamma})^3 d\Gamma$$

By fitting $\ln\left(g^1(\tau)\right)$, $\bar{\Gamma}$ and μ_2 can be acquired, and then converted to the mean size R_Z and the polydipersity index PI as

$$R_Z = \frac{k_B T}{6\pi\eta\bar{D}} = \frac{k_B T}{6\pi\eta} \cdot \frac{1}{\bar{\Gamma}/q^2} \quad \text{(S5)}$$

$$\mathrm{PI} = \frac{\mu_2}{\bar{\Gamma}^2} \quad \text{(S6)}$$

Although valid for polydisperse samples that meet the criterion $\mu_2\tau^2 \ll 1$,[3,6], in this study, the cumulant method was applied to both PS latex nanoparticle samples and vesicle samples for the comparison with NTA.

References

1 Berne, B. J. & Pecora, R. *Dynamic light scattering: with applications to chemistry, biology, and physics*. Dover edn, (Dover Publications, 2000).

2 Stetefeld, J., McKenna, S. A. & Patel, T. R. Dynamic light scattering: a practical guide and applications in biomedical sciences. *Biophys Rev* **8**, 409-427, doi:10.1007/s12551-016-0218-6 (2016).

3 Hassan, P. A., Rana, S. & Verma, G. Making sense of Brownian motion: colloid characterization by dynamic light scattering. *Langmuir* **31**, 3-12, doi:10.1021/la501789z (2015).

4 Merkus, H. G. *Particle size measurements: fundamentals, practice, quality*. (Springer, 2009).

5 International Standard Organization, *Particle size analysis: Dynamic light scattering (DLS)*, in *ISO 22412:2017*. 2017. Geneva, Switzerland: International Standard Organization.

6 Koppel, D. E. Analysis of macromolecular polydispersity in intensity correlation spectroscopy: the method of cumulants. *J Chem Phys* **57**, 4814-4820, doi:10.1063/1.1678153 (1972).

Supporting Information

Supporting Table 1. Size information of the PS latex nanoparticle standards from DLS and NTA.

	DLS		NTA					
			DC		FTLA		Iteration	
Sample	Z-avg	PI	Mean	SD	Mean	SD	Mean	SD
PS 92 nm	91 ± 1	0.037 ± 0.029	94 ± 4	26 ± 3	88 ± 4	12 ± 1	89 ± 4	15 ± 2
PS 269 nm	278 ± 1	0.016 ± 0.009	261 ± 3	47 ± 5	250 ± 3	22 ± 12	250 ± 4	34 ± 7
PS 343 nm	352 ± 3	0.035 ± 0.018	320 ± 3	67 ± 0	294 ± 7	66 ± 12	295 ± 6	63 ± 4

Unit: nm (except PI)

Supporting Table 2. Log-likelihood of the size distributions of PS latex nanoparticles standards estimated by FTLA and iterative methods with respect to the log-likelihood of the size distribution acquired by the direct conversion of the observed tracks of the PS latex standards.

Sample	Δ log-likelihood		Sample	Δ log-likelihood		Sample	Δ log-likelihood	
	FTLA	Iteration		FTLA	Iteration		FTLA	Iteration
PS92	32.94	35.12	PS269	-4.36	9.78	PS343	-37.66	15.94
	41.99	39.66		4.56	7.31		-1.03	22.75
	15.30	26.76		6.75	4.87		-3.07	9.62

Supporting Table 3. Mean size and standard deviation of the size distributions of the POPC vesicle samples from DLS and NTA. DLS results were analyzed by the cumulant method, and NTA results were analyzed by the three different estimation methods, i.e., direct conversion, FTLA and the iterative method.

	DLS		NTA					
			DC		FTLA		Iteration	
Sample	Z-avg	PI	Mean	SD	Mean	SD	Mean	SD
50 nm	91 ± 4	0.089 ± 0.032	100 ± 2	38 ± 7	87 ± 5	24 ± 5	88 ± 5	27 ± 5
100 nm	132 ± 3	0.064 ± 0.035	119 ± 9	45 ± 16	106 ± 5	27 ± 10	108 ± 4	34 ± 10
200 nm	213 ± 4	0.077 ± 0.048	211 ± 6	66 ± 2	193 ± 5	43 ± 2	193 ± 5	49 ± 2
400 nm	458 ± 13	0.291 ± 0.013	329 ± 17	119 ± 13	275 ± 14	146 ± 8	279 ± 14	128 ± 5
FT3	282 ± 4	0.273 ± 0.021	243 ± 12	101 ± 12	212 ± 13	81 ± 8	216 ± 11	95 ± 6
FT5	223 ± 2	0.224 ± 0.022	225 ± 9	121 ± 7	195 ± 6	88 ± 6	196 ± 6	98 ± 6
FT7	185 ± 2	0.197 ± 0.029	201 ± 7	94 ± 2	173 ± 5	64 ± 1	173 ± 5	73 ± 1
FT9	161 ± 1	0.149 ± 0.023	184 ± 8	84 ± 6	160 ± 5	54 ± 3	160 ± 5	61 ± 4
FT11	148 ± 1	0.181 ± 0.024	171 ± 13	90 ± 11	138 ± 8	58 ± 6	138 ± 8	66 ± 7
FT13	131 ± 2	0.168 ± 0.015	158 ± 15	87 ± 14	131 ± 8	54 ± 9	131 ± 8	65 ± 8
FT15	117 ± 2	0.189 ± 0.025	135 ± 4	67 ± 3	113 ± 3	40 ± 2	113 ± 4	48 ± 3
FT17	109 ± 1	0.197 ± 0.021	137 ± 1	72 ± 3	113 ± 1	42 ± 1	112 ± 1	48 ± 2

Unit: nm (except PDI)

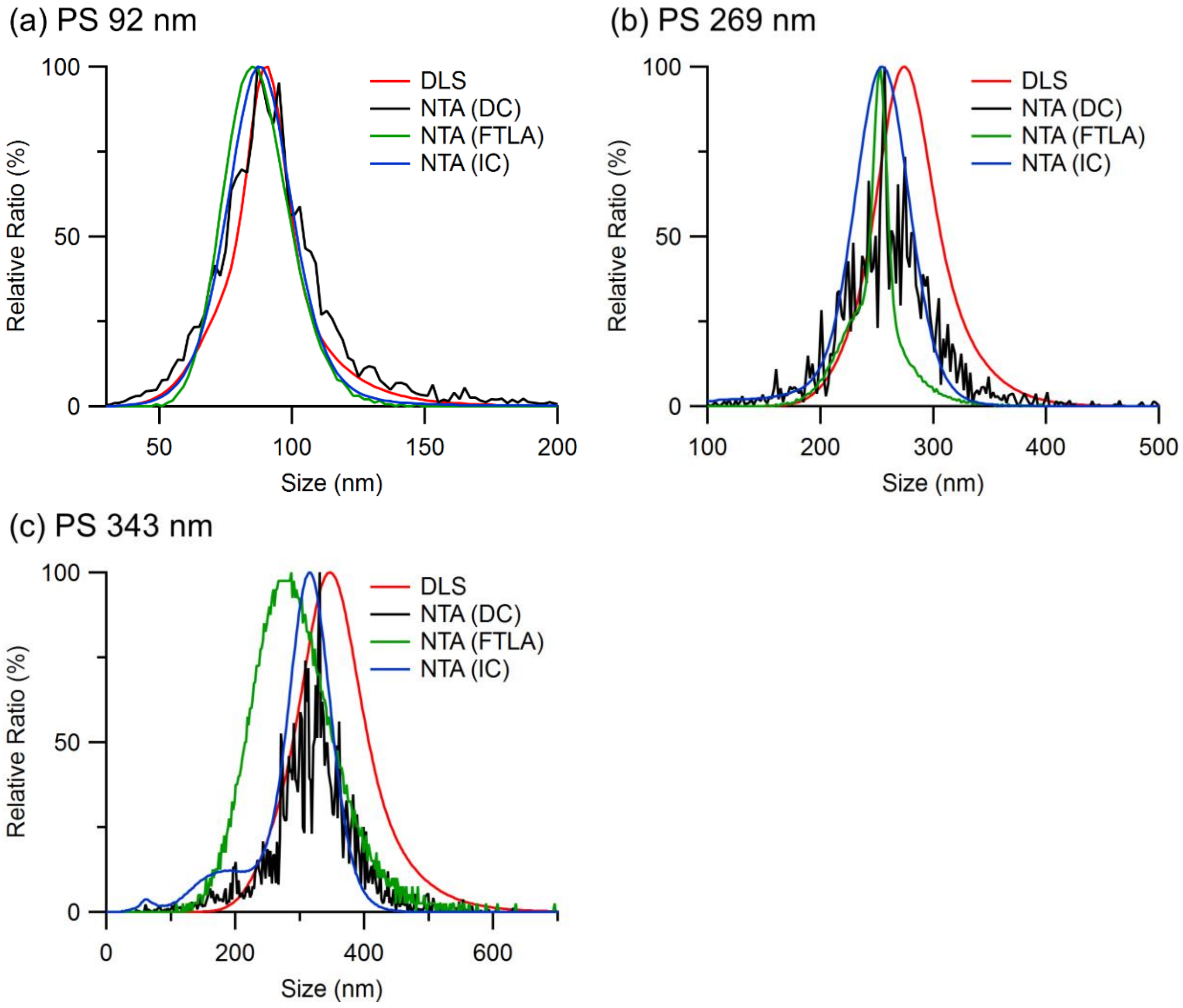

Supporting Figure 1. Size distributions of PS latex standards measured by DLS and NTA: (a) 92-nm, (b) 269-nm and (c) 343-nm PS nanoparticle standards. The size distributions from DLS assume a log-normal distribution acquired from the cumulant method, while those from NTA are from the direct conversion, FTLA and iterative methods. The bin width of the size distributions is in 2 nm.

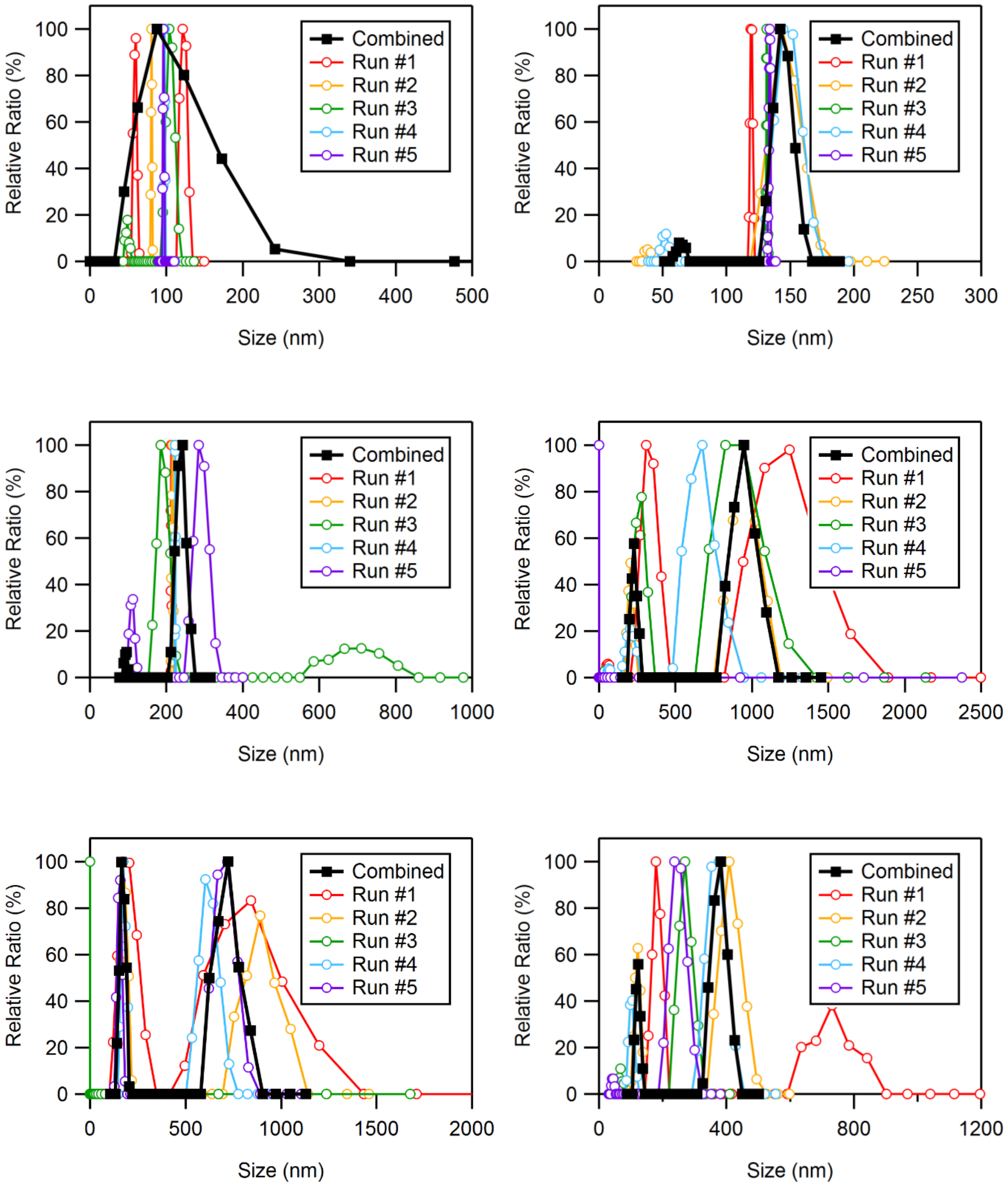
Relative Ratio (%)
Size (nm)
Combined
Run #1
Run #2
Run #3
Run #4
Run #5

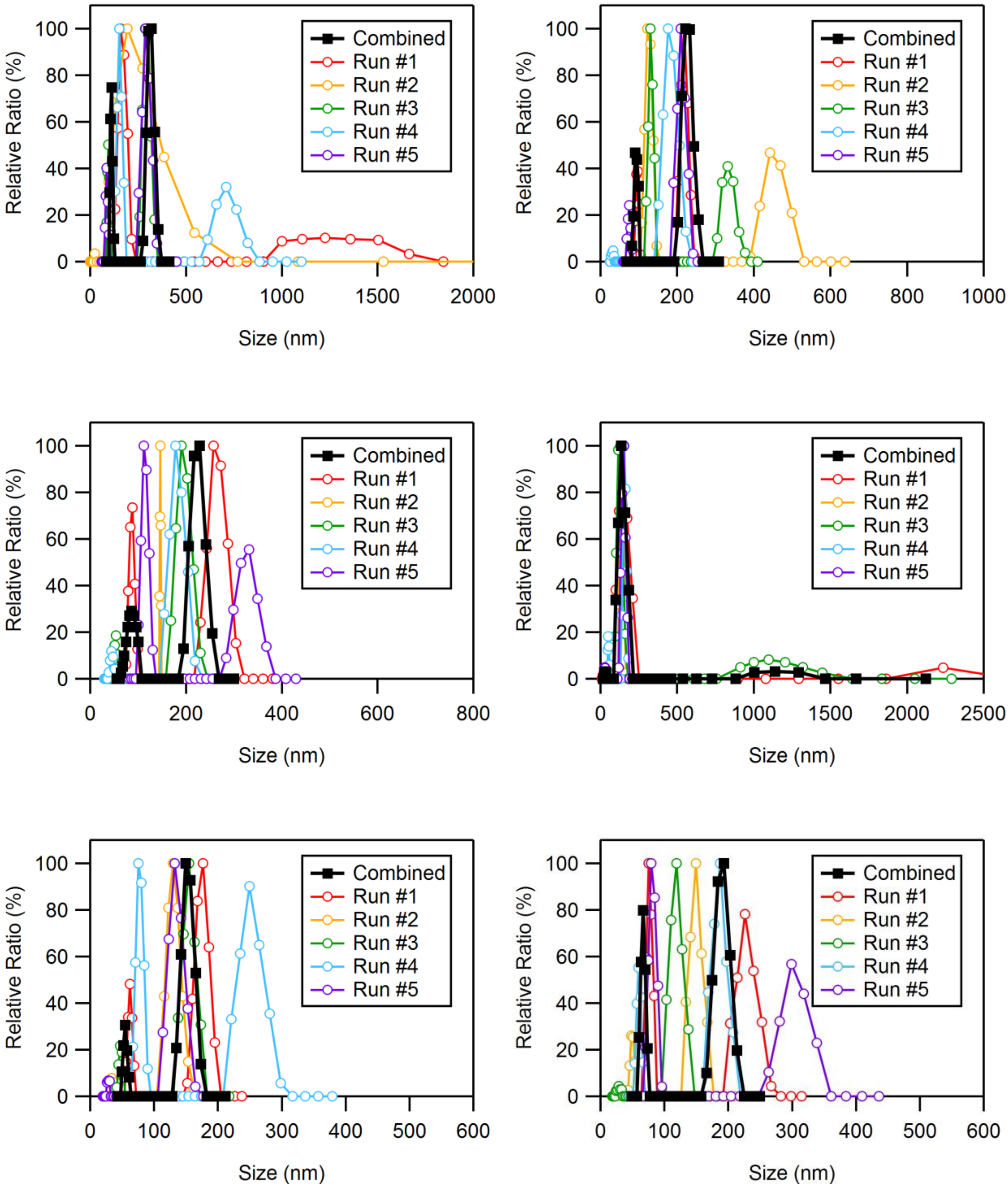

Supporting Figure 2. Size distributions of the POPC vesicle samples prepared in various polydispersity conditions measured by DLS whose distributions were analyzed with the non-negative least square method. The vesicle samples were prepared by extrusion through (a) 50-nm, (b) 100-nm, (c) 200-nm and (d) 400-nm pore filter or by freeze-thaw treatment (e-l) of 3 to 17 cycles before extrusion through a 400-nm pore filter.

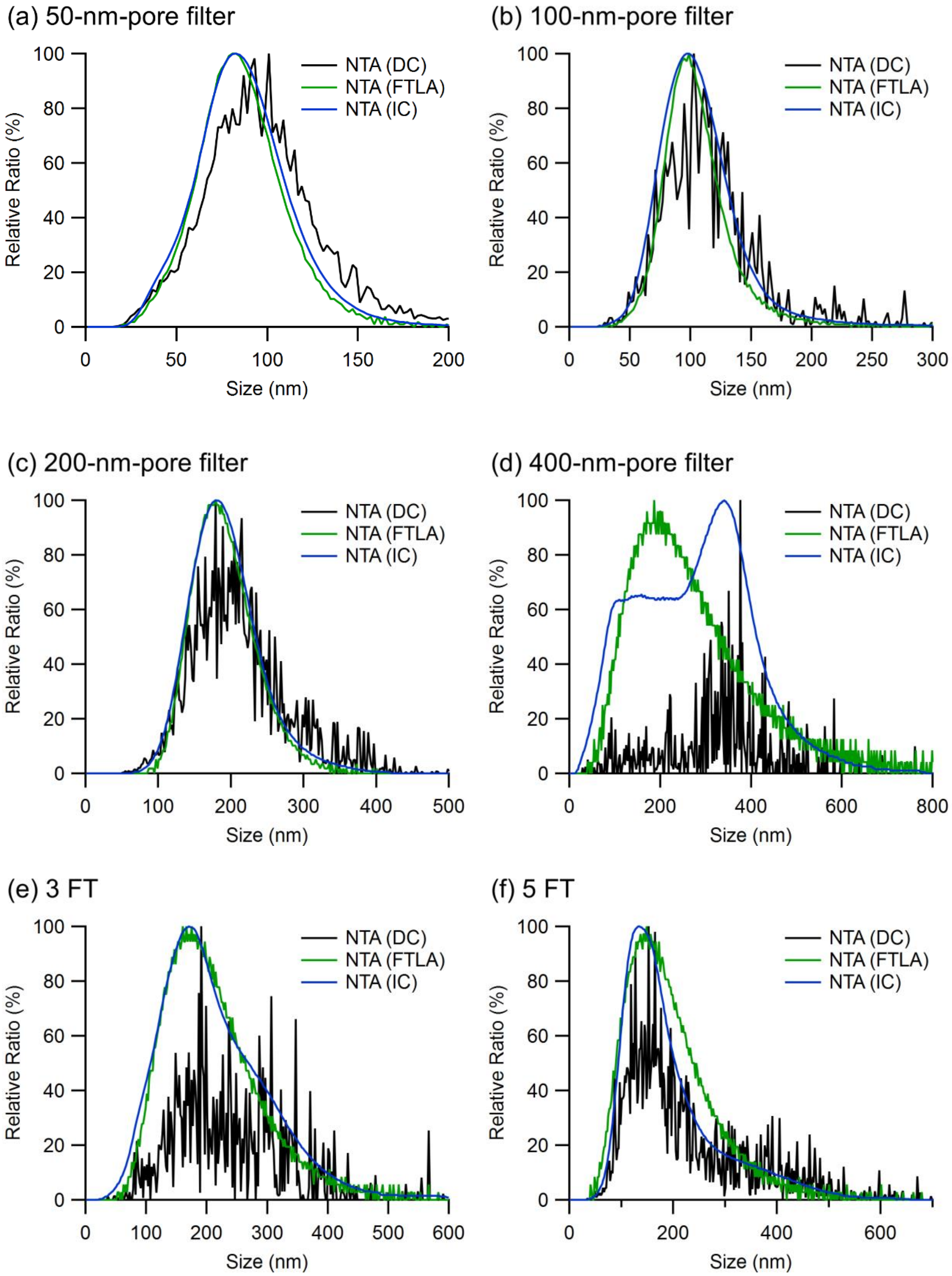

(a) 50-nm-pore filter
(b) 100-nm-pore filter
(c) 200-nm-pore filter
(d) 400-nm-pore filter
(e) 3 FT
(f) 5 FT
NTA (DC)
NTA (FTLA)
NTA (IC)
Relative Ratio (%)
Size (nm)

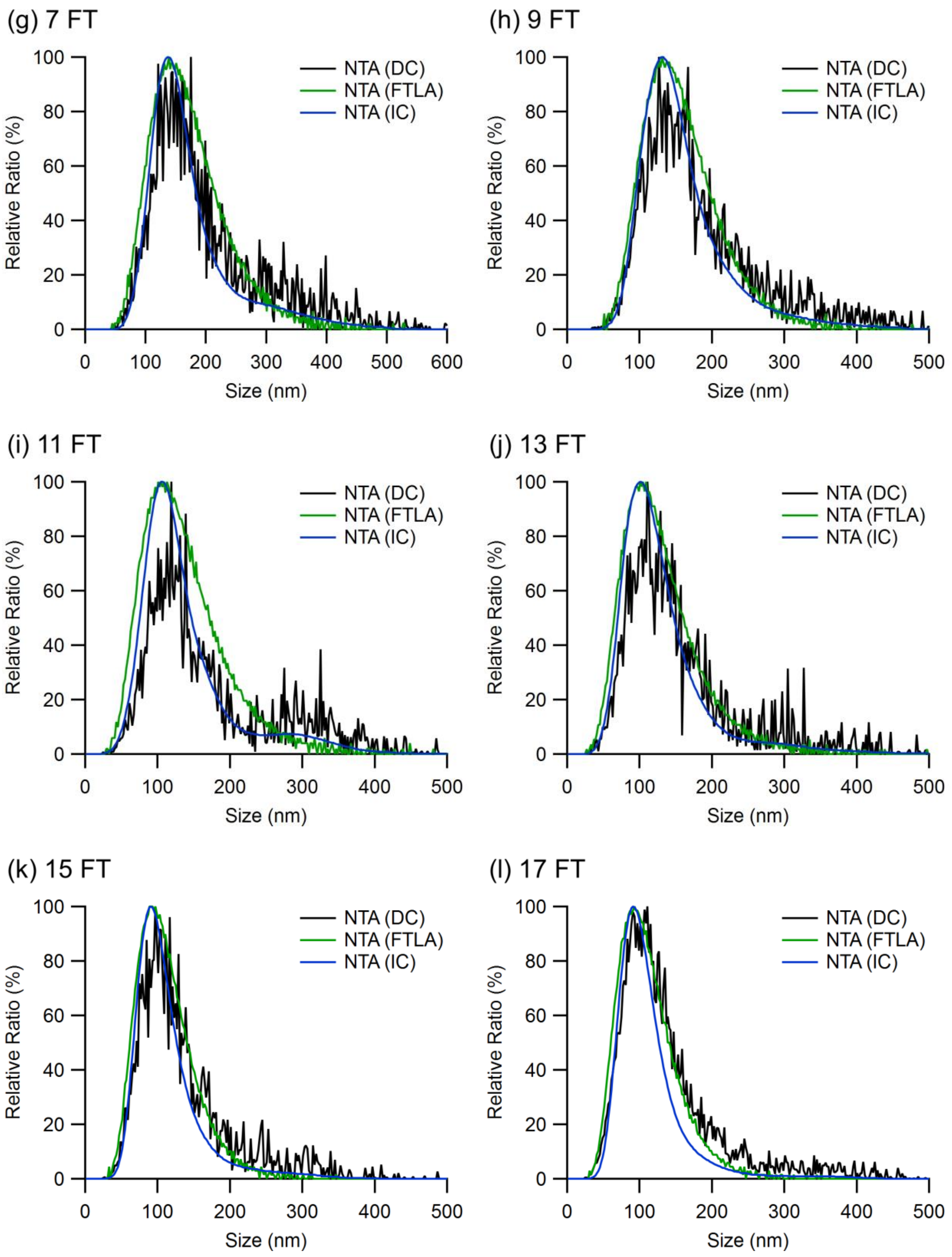

Supporting Figure 3. Comparison of the size distributions of the POPC vesicle samples analyzed by the different size distribution estimation methods for NTA measurements. The direct conversion (black), FTLA (green) and the iterative (blue) methods were applied for the size distribution estimations. The vesicle samples were prepared by extrusion through (a) 50-nm, (b) 100-nm, (c) 200-nm and (d) 400-nm pore filter or by

freeze-thaw treatment (e-l) of 3 to 17 cycles before extrusion through a 400-nm pore filter. The bin width of the size distributions is in 2 nm.
